# Persons living on a disability grant in Mpumalanga province: An insider perspective

**DOI:** 10.4102/curationis.v38i1.1204

**Published:** 2015-09-29

**Authors:** Susanna C.D. Wright

**Affiliations:** 1 Adelaide Tambo School of Nursing Science, Tshwane University of Technology, South Africa

## Abstract

In Mpumalanga province, more than 45 000 persons with disability receive a disability grant. Although research regarding social grants in general and disability grants specifically had previously been conducted from various perspectives, none has been carried out in Mpumalanga and none to explore the impact of the disability grant on the lives of the recipients. The objective of the study was to gain an understanding of the impact of the disability grant on the lives of recipients living in Mpumalanga. The study was conducted as a contextual, exploratory and qualitative study. The target population was persons with a disability receiving a disability grant. Data gathering was conducted in October 2010 using a semi-structured interview technique. The data were analysed in terms of the social and economic impact of the disability grant in the life of the participant. A combination of three qualitative data analysis methods was used to analyse the data. The qualitative findings indicate that although it is an individual grant, the disability grant was used to support the whole family and was frequently the family's only income. Food, clothes and electricity was most frequently bought with the disability grant. Food often did not last for a month. The families were living precariously and any crisis, for example lapsing of the grant, would result in hunger and desperation as a result of their complete dependence on the disability grant. Without insight in how people live their lives, registered nurses may give health education to patients that they cannot implement, perpetuating the burden of disease in South Africa.

## Introduction

The South African Welfare System is premised on full employment (Nattrass 2006:3), implying that only those unable to work, too young, old or sick qualify for social assistance despite lack of employment opportunities. South Africa is characterised by extreme inequality, with high rates of unemployment and consequent absolute levels of poverty (Leatt & Budlender 2007). Mitra (2008) describes the relationship between disability and poverty as a ‘vicious cycle’. Disability adds to the risk of poverty, and conditions of poverty increase the risk of disability; the result of the cycle is that people with disabilities usually are the poorest of the poor. From a financial point of view, Samson et al. (2002) report that in South Africa, the social security grants reduce the income gap by approximately 23%. The focus of this article is the disability grant which is a monthly income support for adults who are in financial need and who have a disability which prevents them from being able to support themselves (Western Cape Government 2014).

It is imperative that health care professionals and social workers have some understanding of the lives of a person who has to survive on a disability grant. Registered nurses or social workers in health care settings or government and municipal service departments respectively, frequently have to care for to persons living on a disability grant as the only income in a household. A person's socio-economic background has a direct influence on their health related needs and ability to access health and follow a treatment plan. Frequently, a health consultation and treatment plan does not have the intended outcome, resulting in chronic disease and unforeseen new health problems such as multidrug or extreme drug resistant tuberculosis or the frequent measles outbreaks. The registered nurse has to take cognisance of the context of the person cared for when giving health education. If the person cannot adhere to advice because of a lack of funds, health care workers are contributing to the burden of disease in South Africa. Although the current study was conducted in Mpumalanga province, the insight gained would be applicable anywhere in South Africa.

From a legislative perspective, the Constitution of South Africa (South Africa 1996) protects a person's right to social security. Section 27(1) (c) of the Constitution provides that everyone has the right of access to ‘… social security, including, if they are unable to support themselves and their dependents, appropriate social assistance.’ Social assistance in South Africa consists of seven non-conditional and non-contributory cash grants of which the disability grant is one. According to the Department of Social Development, disability grants are available to adult South African citizens who are incapacitated and unable to work as a result of illness or disability (Department of Social Development 2004). The disability grant is the only social assistance adults of working age can access through the South African Welfare System.

This study explored the impact of the disability grant on the lives of recipients living in Mpumalanga.

## Research problem

In 2001, Mpumalanga was home to 7.0% of the South African population (Statistics South Africa 2001). In 2005, the figure reached 3.5 million with the unemployment rate at 37.0% according to the expanded definition, whilst the human development index was 0.60 and the Gini coefficient 0.64 (Mpumalanga provincial government 2007:1). In 2007, more than 133 000 persons received a disability grant of R1200 per month with the majority (93.2%) from black socio-cultural groups (Statistics South Africa 2007). If the grant is the only income for a household of six persons on average, how do they survive for a month? The research question for the study was: how do households of six persons on average live if the disability grant is the only income of the family?

## Purpose and objective of the study

The purpose of the study was to be able to inform policy regarding disability grants and, in doing so, improve the lives of persons receiving a disability grant in Mpumalanga.

The objective of the study was to gain an understanding of the impact of the disability grant on the lives of recipients living in Mpumalanga.

## Definitions of the concepts

The following definitions were accepted for the study:

**Social grant:** According to the *Social Assistance Act* (Act 13 of 2004), a social grant means a child support grant, a foster care grant, a disability grant, an older person's grant, a war veteran's grant and a grant-in-aid (South Africa, Act 13 of 2004).

**Social assistance:** Social assistance is state-provided basic minimum protection to relieve poverty, essentially subject to qualifying criteria on a non-contributory basis (Committee of Enquiry into a Comprehensive System of Social Security for South Africa 2002:36). The disability grant falls into the category of social assistance.

**Disability:** Disability, in the context of the study, means being older than 18 years of age and unable to work because of a mental or physical disability. Seven types of disability are recognised, namely, sight, hearing, communication, physical, intellectual, emotional and multiple disability (South Africa, Act 13 of 2004).

**Impact:** Impact, in terms of the study, is defined as the social and economic influence of the disability grant on the life of the recipient from the perspective of the recipient.

## Materials and methods

### Design

The study was contextual, exploratory and qualitative, in investigating the impact of the disability grant on the life of the recipient living in Mpumalanga from the perspective of the participant. The design was suitable for the study as the focus was on the participants’ perceptions of how they live their lives.

### Research setting

The study was conducted in the three districts of Mpumalanga, namely Gert Sibande, Nkangala and Ehlanzeni. In each district, an urban and rural setting was purposefully chosen to investigate the impact of the grant in a variety of settings. Table 1 presents the chosen rural and urban municipalities.

**TABLE 1:** Rural and urban municipalities chosen per district.

District	Rural	Urban
Gert Sibande	Albert Luthuli	Pixley ka Seme
Nkangala	Dr JS Moroka	Victor Khanye Local Municipality
Ehlanzeni	Bushbuckridge	Mbombela

### Population

The target population was the recipients of disability grants in Mpumalanga. As there may be a difference in the impact on the lives of the recipients in the various districts, disability grant recipients of all three districts were included and the sample size of each district was saturated independently. The municipalities in each district were classified as predominantly urban or rural and one of each was chosen conveniently. In each of the chosen municipalities, the South African Social Security Agency (SASSA) prepared a list of disability grant recipients in the municipality. People on the SASSA lists were contacted and invited to participate. Because of the logistical challenges of gathering data in such a vast province, specifically in the rural areas, a decision was taken to conduct 30 semi-structured interviews per district, 15 in each municipality and to do the analysis after data gathering. If it was found that the data were not saturated, additional interviews would have been carried out.

The sampling method was purposive and convenient. The inclusion criteria were: a recipient of a disability grant from Mpumalanga; willingness to participate in the study; both male or female; and living in the chosen urban or rural municipality in the three districts of Mpumalanga.

Saturation was achieved in all six municipalities at the 10th to 12th interview.

### Data gathering

A semi-structured interview was used to gather data and generate a self-report in October 2010. The data-gathering instrument used was an interview schedule which consisted of two sections, including a demographic profile of the participant which was extensive, containing information regarding:

- The location of the participant (district, municipality, urban or rural residence).
- Type of disability.
- Socio-demographic information (gender, age group, population group, marital status, housing, employment status, educational level and a profile of the number of persons living in the house as well as the children in the household).
- Grant information (permanent or temporary status of the grant, the year the grant was first received, and the amount).

The interviewer completed the demographic data on the prepared form and then audio-taped the interview with permission. Rich data were gathered by direct questioning of people to report on their own experiences. The section exploring the impact of the disability grant included several open-ended questions and focused on the collection of the grant, the use of the grant in the household, and decision-making regarding the spending of the grant.

## Data analysis

Data analysis commenced after the interviews were completed. A combination of three qualitative data analysis methods was used. A template analysis style (Polit & Beck 2008:508) was combined with content analysis using open coding (Creswell 2003:192) and quasi-statistics (Polit & Beck 2008:517). A simple frequency count of how many times a phenomenon occurs provides the researcher with more insight and made a stronger conclusion possible.

## Trustworthiness

Trustworthiness was established according to the strategies promoted by Lincoln and Guba (1985). Trustworthiness is a method of establishing rigour in qualitative research without sacrificing relevance. The principles of credibility, dependability and transferability were applied. The fieldworkers were socially and culturally congruent with the community members of the district where they gathered data. Member checking was achieved before the interviewer left the participant. An audit trail was developed to enable checking of the findings against the raw data of the study.

## Ethical considerations

Brink, Van der Walt and Van Rensburg (2006) identified several ethical principles which were applied in the study.

Informed consent was obtained from participants before participation in the study. Participants were informed about the freedom to participate or not, and of their right to withdraw from the study at any time; assurance of anonymity and confidentiality, openness and privacy was provided.

Although the respondent's name was used during the interview to create rapport and cultural requirements, anonymity and confidentiality was ensured by numbering the transcribed interviews sequentially and removing the names during the transcription. Permission included the use of a digital audio recorder and taped material which was erased on completion of the data analysis by the researcher. This was carried out as a final measure to ensure anonymity of the participant.

The interview was conducted in a language the participant was comfortable with, although the study was reported in English. Fieldworkers, conversant with the local language, were trained and supervised to gather the data for the study.

Because of the personal nature of the study, emotional discomfort may be experienced. Should counselling be necessary, arrangements were made through the Department of Social Development for counselling

Approval was obtained from the Ethics Committee of the Departments of Health and Social Development in Mpumalanga.

## Results

### Demographic profile

A total of 90 participants were interviewed. The demographic profile for the urban municipalities is presented first (Table 2). In Pixley ka Seme and Mbombela, the majority of the participants had a physical disability; in Victor Khanye local municipality, emotional disability was most frequent (*n* = 7). In Pixley ka Seme and Mbombela, more females (*n* = 11 and *n* = 10) were included, whereas in Victor Khanye, more males (*n* = 10) were included in the sample. The modal age group was 41 to 50 years with 19 participants for the combined group, followed by 15 participants in the 31 to 40 years age group. For all three urban municipalities, the majority of the participants (32 of 45) was never married. A third of the combined urban group (*n* = 16) never had any schooling, followed by 23 participants who achieved less than grade 12. The Pixley ka Seme sample was less literate compared to the Victor Khanye and Mbombela samples. The number of children per household varied between none in 10 households to five in four households: the modal number of children per household being two (15 of 45 households). In each of the urban municipalities, the ages of the majority of the children tended to be younger (Pixley ka Seme 17 of 30 children, Victor Khanye 18 of 28, and Mbombela 16 of 30). The majority of the children (46 of 64) were still in primary school.

**TABLE 2:** Demographic profile of recipient of the disability grant and household members in the urban (*n* = 45) and rural municipalities (*n* = 45).

Recipient	People living in house		People supported by grant	
		Number	Average	Number	Average
Pixley ka Seme	0	5.5	2-11	5.1
	11	-	-	-
	3	-	-	-
	1	-	-	-
Victor Khanye	0	5.2	2-9	5.2
	6	-	-	-
	9	-	-	-
	0	-	-	-
Mbombela	2	4.9	1-8	3.7
	9	-	-	-
	4	-	-	-
	0	-	-	-
Albert Luthuli	0	5.6	2-9	5.2
	6	-	-	-
	8	-	-	-
	1	-	-	-
Dr JS Moroka	2	5.1	1-7	3.6
	4	-	-	-
	8	-	-	-
	1	-	-	-
Bushbuckridge	0	6.4	2-9	5.8
	6	-	-	-
	9	-	-	-
	0	-	-	-

In terms of the rural municipalities, the type of disability varied within each municipality. In Albert Luthuli, the majority of the sample had a physical disability (*n* = 12), in Dr JS Moroka emotional disability (*n* = 7) and in Bushbuckridge, multiple disability (*n* = 8). In all three municipalities, both males and females were included in the study with more males (*n* = 24) than females (*n* = 21). The modal age group was 21 to 30 years (24 of 45) followed by a 51 to 60 years age group, but in Albert Luthuli, all the participants were in the 21 to 30 years age group. As was the case with the urban municipalities, the majority of the rural sample (*n* = 36) was never married. Seven of the 45 households had no children as part of the family. The modal number of children per household was two (*n* = 14). Nine households had three to four children to support and only two households supported five children. There were more children in the combined rural sample (*n* = 98) when compared to the combined urban sample (*n* = 87). A large proportion (43 of 51) of the children was still in primary school. In terms of the age of the children, more than half of the combined rural children sample (54 of 98) were 10 years of age or younger. The modal age group was 11 to 15 years in 28 children.

From Table 2, the average number of people per urban household was 4.9 for the combined urban sample. Mbombela had the smallest average number of people living in a house (3.7), followed by Victor Khanye (5.2) and lastly Pixley ka Seme with 5.5 people per household. The importance of these figures is underscored by the number of people per household, supported by the disability grant. In Pixley ka Seme, the average number of people living in a house supported by the grant was 5.5 and 5.1, in Victor Khanye 5.2, compared to Mbombela where the number supported was lower (3.7 supported and 4.9 living in the house).

From the rural group, the average number of people living in a house was 5.6 (Table 2). The highest average was recorded for Bushbuckridge with 6.4 persons per house. The average number of persons supported by the disability grant was 4.7; the lowest was in Dr JS Moroka with 3.6 persons supported per disability grant and the highest in Bushbuckridge with 5.8. The range of people supported by the disability grant was 2 to 9 for both Albert Luthuli and Bushbuckridge, with slightly less at 1 to 7 for Dr JS Moroka.

In both the urban and rural group, the majority of the combined urban sample received a permanent disability grant.

## Qualitative findings of the study

Several themes were developed to describe the impact of the grant in the life of the recipient. The themes include the use of the grant for living necessities, multiple grant related income per household, and use of the grant for children's education and health related expenses. In all the municipalities, the grant was used as cash, even for some of the grant recipients who had a bank card to pay for commodities.

### Necessities for living

In terms of living necessities, food and clothing were first on the list in every municipality. Food is sometimes bought on credit to be paid at the end of the month. Several participants reported that food accessed was insufficient to last for the month, specifically in families where the grant recipient was the only source of income for the household. They explained: ‘The food is not enough. It has never happened that the food is enough, it is too little.’

The disability grant was never considered for personal use only, but considered as an income for the household and spent as such. The total dependence on the disability grant was described as follows:

‘I buy food, clothes for the child and I help the other children here at home with their education. We use the grant but it is not enough for the whole month, you find that the money is finished in the middle of the month and we just have to wait for the end of the month. I just sit and accept because I do not have parents and my brother is sick, he has TB so that he also has to go for treatment.’

‘With the money, I buy groceries and then I am left with nothing. I can’t even buy things to wash, it is too little.’

All participants bought clothes for their family and themselves. It was never possible to buy all that was required in one month. Participants planned their expenditure, and would budget for clothes this month and blankets the next month. ‘Sometimes I can buy clothes but it is only after a long time because my priority is to buy food [*for seven people*] at the house’ and ‘I don’t know when did I last bought clothes, when I was still working the money is too small.’

Furniture was purchased with the grant but always on an instalment plan or a lay-bye. A wardrobe was frequently bought: ‘With the wardrobe I paid bit by bit until I finished.’ Blankets were always a priority and bought from Asian entrepreneurs who allowed the participants to take the blankets and pay them off ‘bit by bit.’ Some however, could not buy any furniture: ‘There are needs, the house things. I cannot do them because the money is not enough for me to be able to get furniture.’

### Water and electricity

Although not all participants paid for water and electricity, the majority did. One participant acknowledged they were connected illegally: ‘That is my problem I end up connecting illegally.’ No one reported using coal for cooking but some used wood when the electricity ran out. The amounts paid varied from R30.00 to R300.00 per month. In some instances, paying for water was a problem:

‘Water? No we do not pay we fetch water from far very far. Look for this whole week there has been no water we drink water from the river. Last December children died in that river, they died in that river and look now we drink the same water.’

‘What can you do with the water I do not pay as I am expected but with the electricity I won’t because they will terminate it?’

‘When I have just been paid, I buy for R50 and when it is finished, I try to buy for R10.’

‘Water, these days we suffer with water.’

### Funeral cover

Less than half of the participants paid for funeral cover ranging from R80 to R180 per month. Participants knew of the importance of providing funeral cover but were not always able to do so. The conflict of knowing but being unable to do so is clear from the following participant's explanation: ‘I used to pay Maiteko (social burial club) but then I was not able to give my children pocket money for school so I left it.’

No participant reported being able to save any money.

### Transport

In terms of transport, participants mostly walked to their destination, using a taxi infrequently.

### Multiple grant related income per household

In a household where there were more than one recipient of a grant (disability grant, old age pension and child grant), the grants were used in combination. Each person retained the right to spend their own grant, but if there was a need, resources were combined, for example:

‘I do a lot because if they need a shirt like the other one who needed shoes we had to add money and buy the shoes so that he can continue and study.’

‘The grant helps me here at home. My mother receives a grant as well, she buys food and when I get mine, I also give her money. Even the children's grant, it helps us a lot.’

### Children's education

In most of the six municipalities, more than two-thirds of the participants had school-going children and in all instances, the disability grant was used to support the children's school fees, to buy uniforms or to pay for additional trips. Participants explained as follows:

‘My younger sibling goes to school. I pay school fees and uniform. He sometimes tells me that he needs school shoes, even if I buy, it will just be the shoes.’

‘I buy clothes for the school going children but it [*disability grant*] is not enough because you will find there is much costs then.’

‘In January they will come and say they need school fees, how can I afford R300?’

### Health related expenses

Although participants were usually careful to have money available should they need medical care or assistance, this was not always the case. One participant said: ‘In that case, I have to go house to house borrowing money because mine is finished.’ However, in most instances, participants budgeted for their health care.

‘With the grant, when I receive the money I calculate and say with this one I will buy this and that, and the one that I don’t use I save for whenever I need money let's say I become sick I can withdraw and use it.’

‘We just force things but the money is not enough like now I am alone, it is because I buy expensive things because I am diabetic. They complain that I do not eat the right food. I am not able I buy this thing this month and the other thing the month I alternate because I cannot buy all at once but it has to go together, like with Margarine I have to eat Elite and the juice I can’t drink any.’

‘Ahhhhh, how will I buy? It is true as you say and I have diabetes and arthritis with high blood and this things that I eat in the clinics they measure them and say you need to eat this and that how will I eat such things because I don’t have money I just eat anything.’

With regard to follow-up clinic or hospital appointments, all the participants reported being meticulous. The majority received their medication from the local clinic. Only one person, who had a long-term physical disability, explained she did not use the formal health care system as a rule because of a lack of funding.

### Using a loan to supplement income

Some participants reported that they would get a loan at an exorbitant interest rate. The repayment would deplete their disability grant to such an extent that they would have to borrow money again the next month: ‘There is this other lady who is a loan shark, I go to her and ask for a 12.5 kg [*maize meal*]. When the money comes back it has interest its equal R65.’

## Discussion

The most frequent type of disability, for both the urban and rural group, was physical. In some instances the deduction, from the narratives, was that the underlying disease was HIV and AIDS related, but a large group also mentioned arthritis and orthopaedic related skeletal health problems. Support for the non-infectious origin of the disability is the age distribution of the urban group. More than half of the urban group (27 of 45) were older than 41 years of age, but in the rural group, only 14 of the 45 participants were in this age category.

The use of transport was infrequent, mostly for health related visits and always budgeted for carefully. The majority of the participants used the local clinic or hospital for health care as it was free of charge. Specialists were only used after a referral from the local health practitioner. There was one exception. In Albert Luthuli, although participants would use the clinic if they felt really ill, they would, although expensive, go to a private doctor, at R200.00 to R250.00 per consultation. The reason for not trusting the local health care system is unclear.

In both urban and rural samples, the majority of the participants (39 of the 45) did not achieve grade 12 level of education. The proportion of participants not receiving any schooling was high in the urban sample (16 of 45) compared to 3 of 45 participants in the rural sample. There were also variations in the urban sample with 10 of the 15 participants in Pixley ka Seme not having any schooling compared to 1 of 15 Mbombela participants. The high level of functional illiteracy seems to have an influence on the way information is disseminated to disability grant recipients.

It was unclear whether all eligible children received a child grant and there was no specific question to determine if they should. The number of children younger than 10 years old were high in both rural (54 of 98) and urban (51 of 87) households. Additionally, in the rural sample, there were 11 households with 4 or 5 children per household and 8 such households in the urban sample. If the disability grant recipients were as uninformed regarding child grants as they were regarding their own disability grant, it is quite possible that many of the households did not receive the financial support they were legally eligible to.

Families frequently used the disability grant to pay school fees and to buy school clothes. Although, being an individual grant and on paper supposed to be used for the disabled individual, in practice this was not the case. Support for this statement is found in the number of persons supported by the disability grant in a household. In total, the disability grant issued to 90 persons supported 185 children, most of who were still in primary school. Research should be conducted to inform policy with regards to using a disability grant to pay school fees, where it is the only income in the family.

Saving for a future problem was almost non-existent in the combined urban and rural sample. It was not as though saving was not considered important, but it was mostly impossible. The most frequent method of saving was participating in a stokvel group where a number of women would contribute a monthly amount, usually about R100.00, and use the cash when it was her turn to buy either clothes or a piece of furniture. A large group of the participants, except for the Mbombela sample, paid monthly into a funeral society which was also community based and in some instances would only supply a coffin, not the social occasion accompanying a funeral. The understanding gained was that it was the sensible thing to do as we would all die some day and the person should not be a burden to the family post-mortem.

## Limitations

The following limitations of the study are noted:

- Six different field workers were used to gather the qualitative data. Although they were trained twice, depth of probing was, in some instances, lacking. The lack of depth may, however, be offset by the culturally congruent nature of the fieldworkers in the three districts.
- Because of the qualitative nature of the study, some aspects were raised but not explored in depth.
- The data was self-reported and, as such, social desirability bias may have influenced it.
- There is a lack of literature to support or contrast the findings of the current study.

## Recommendations

Several recommendations emanated from the evidence generated in the study.

Any health care-related prescription should take the context of the lives of this group of people into consideration.

In a household where the disability grant is the only income, exemption of paying for essential services such as electricity and water should be considered.

The reality of school going children depending on the grant, the majority of whom are still in primary school, should be accepted. Several recommendations regarding school going children emanated from the study:

- Although some participants reported that their children were eligible and received a child grant, it was unclear whether all eligible children received a grant. An investigation is required.
- Children living in a household where the disability grant was the only income for a livelihood should be exempted from paying school fees.
- Many of the children in the household were orphans and it was unclear whether the children were receiving appropriate financial assistance, or whether the disability grant recipients were knowledgeable regarding the foster care grant. Support from the Department of Social Development is required.

## Conclusion

The disability grant fulfils a life-saving function in the family where a person with a disability lives. The influence is profound and literally a matter of life and death, frequently being the only income in the family. The group most assisted by the disability grant is children and specifically children less than 10 years old. Without an understanding of how people live in their environments, health care professionals may not understand why health education is not followed. A feeling of frustration and desperation could result if the person living on a disability grant cannot comply with the health education as a result of a lack of funds. Health promotion must be considered carefully and contextualised for the individual, because without insight into the lives of our patients, the result may be harming instead of improving health.
